# Development of Next-Generation Vaccines

**DOI:** 10.3390/vaccines10020274

**Published:** 2022-02-10

**Authors:** Takashi Imai

**Affiliations:** 1 Department of Microbiology, Saitama Medical University, Saitama 350-0495, Japan; t_imai@saitama-med.ac.jp; 2 Department of Infectious Diseases and Host Defense, Gunma University, Maebashi 371-8511, Japan

The battle between pathogens and hosts began on primitive Earth, and will probably continue forever. New infectious diseases in humans, such as COVID-19, AIDS, Ebola hemorrhagic fever, and SFTS (severe fever with thrombocytopenia), are appearing and spreading. Tuberculosis, which once appeared to have been suppressed in some countries, has begun to spread again (re-emerging infectious diseases). In addition, the spread of tropical/subtropical infectious diseases, such as Malaria, Dengue fever, Zika fever and West Nile disease, is feared due to global warming and globalization. Emerging and re-emerging infectious diseases are also seen in animals (foot-and-mouth disease and West Nile disease, etc.). Zoonosis and livestock-derived food poisoning caused by eating contaminated meat and eggs, are also important; thus, in a way, veterinary medicine might contribute not only to animal but also to human health.

Since the first vaccine developed by Edward Jenner, “The father of immunology”, the vaccine has proven to be one of the greatest innovations, saving countless lives. The vaccine is a highly powerful weapon against pathogens by inducing strong immunity and immunological memory; for example, smallpox has now been eradicated thanks to the smallpox vaccine. Additionally, now, the vaccine is not only applicable for the prevention of infectious diseases, but also to prevent or treat cancers/tumors. The ancient vaccine method called “variolation” was developed in India in B.C. 1000. It goes without saying that the wisdom and efforts of our predecessors are the basis of the prosperity of modern society. Now is the time that vaccine scientists in this age can contribute to a better future by developing next-generation vaccines against pathogens and cancer for humans and animals, just as our ancestors did.

In this Special Issue, we will share novel results from Austria, Canada, China, Japan, Korea, Mexico, Netherlands, Russia and Taiwan regarding next-generation vaccines and basic techniques/knowledge for the next step.

Next-Generation Vaccine Development related to Bacteria and Toxins.

Bacille Calmette–Guérin (BCG) is a vaccine against tuberculosis (TB). To overcome the disadvantage of BCG due to the administration route, Vasilyev et al. developed the mucosal vaccine based on the recombinant attenuated influenza vector (Flu/THSP) co-expressing TB10.4 and HspX proteins of *Mycobacterium tuberculosis* [1]. BCG prime Flu/THSP boost immunization protected mice from severe lung pathology via *Mycobacterium tuberculosis*. Their vaccine strategy enhanced T-cell responses.

*Shigellae* and enterotoxigenic *Escherichia coli* (ETEC) cause diarrheal disease. Harutyunyan et al. developed the Shigella and ETEC combination vaccine (ShigETEC) based on a live attenuated Shigella strain [2]. The ShigETEC vaccine conferred serotype-independent protection in a mouse shigellosis model, and induced bacteria-specific IgG and IgA antibody production. Additionally, this vaccine is in Phase 1 study and might be a promising oral vaccine candidate against Shigella and ETEC.

Kim et al. developed a vaccine against fowl typhoid caused by *Salmonella enterica* that was safer than the live attenuated vaccine [3]. They deleted the rfaJ gene of SG9R (live attenuated vaccine strain) to reduce the risk of reverting to a pathogenic phenotype and causing disease. Knockout strain Dtx-9RM reduced the suppressed adverse side effect but showed effectiveness against challenge infection with the field strain. This vaccine might be applicable to the prevention of chicken fowl typhoid.

Su et al. developed a biofilm vaccine that was created using *Lactcoccus garvieae* on the chitosan particle. The biofilm vaccine was orally administered to fish “Mullet” to prevent *L. garvieae*; it induced a greater phagocytic response and a higher production of pro-inflammatory cytokines than a whole-cell vaccine. The biofilm vaccine confers better protection against the challenge infection of *L. garvieae* than the whole-cell vaccine [4]. Effective feed vaccines are preferred by aquaculture companies.

Michiels et al. developed a new assay for the quality control of tetanus toxoids [5]. Animal tests were required for the toxoid vaccine to confirm the batch quality. This assay is based on a combination of cathepsin S digestion and tetanus toxoids to mimic the degradation process inside antigen-presenting cells and to quantify peptides by using liquid chromatography–mass spectrometry. This assay enabled the replacement of animal testing with an in vitro experiment.

Next-Generation Vaccine Development related to Viruses.

Viruses are currently used as a key tool in many viral vectors and licensed vaccine products and gene therapy. Extracellular vesicles (exosomes and macrovesicles) share many features with enveloped viruses. Minh and Kamen reviewed the characteristics and similarities of EVs and enveloped viruses, as well as the processing steps and analytical techniques currently implemented to manufacture and document viral vector and vaccine products [6].

Coxsackievirus causes hand, foot and mouth disease. Chen et al. optimized the condition of Vero cell culture in a disposable bioreactor [7]. The Vero cell is utilized for the production of virus particles, and they aimed to develop an inactivated Coxsackievirus A16 vaccine. This technology for efficiently culturing mother cells can be the basis for the development of next-generation vaccines.

Next-Generation Vaccine Development against Cancer/Tumor.

Imai developed a DNA vaccine against cancer and showed the effect of immunological differences in N-terminus amino acid on the target antigen [8]. In order to induce an antigen response via a vaccine, protein is needed; however, the induction of antigen-specific cytotoxic T cells needs another step, which is antigen degradation and the presentation of MHC class I molecules. His study showed that the small difference in the N-terminus induced a big difference in the outcome.

Martinez-Perez et al. developed a next-generation vaccine that enables the destruction of the cancer cell and induces immunity against human papillomavirus (HPV). Their vaccine is an oncolytic adenovirus vector expressing the fusion antigen of HPV-16 E7 and adjuvant SA-4-1- BBL [9]. HPV-16 E7 is a tumor-associated antigen derived from an HPV-induced tumor. This vaccine selectively lysed the cancer cell in vitro and showed anti-tumor efficacy in vivo.

I believe the articles of this Special Issue constitute one step closer to a better future.

## References

[1] Vasilyev K., Shurygina A.P., Zabolotnykh N., Sergeeva M., Romanovskaya-Romanko E., Pulkina A., Buzitskaya J., Dogonadze M.Z., Vinogradova T.I., Stukova M.A. (2021). Enhancement of the Local CD8(+) T-Cellular Immune Response to Mycobacterium tuberculosis in BCG-Primed Mice after Intranasal Administration of Influenza Vector Vaccine Carrying TB10.4 and HspX Antigens. Vaccines.

[2] Harutyunyan S., Neuhauser I., Mayer A., Aichinger M., Szijarto V., Nagy G., Nagy E., Girardi P., Malinoski F.J., Henics T. (2020). Characterization of ShigETEC, a Novel Live Attenuated Combined Vaccine against Shigellae and ETEC. Vaccines.

[3] Kim N.H., Ko D.S., Ha E.J., Ahn S., Choi K.S., Kwon H.J. (2021). Optimized Detoxification of a Live Attenuated Vaccine Strain (SG9R) to Improve Vaccine Strategy against Fowl Typhoid. Vaccines.

[4] Su F.J., Chen M.M. (2021). Protective Efficacy of Novel Oral Biofilm Vaccines against Lactococcus garvieae Infection in Mullet, Mugil cephalus. Vaccines.

[5] Michiels T.J.M., Tilstra W., Hamzink M.R.J., de Ridder J.W., Danial M., Meiring H.D., Kersten G.F.A., Jiskoot W., Metz B. (2020). Degradomics-Based Analysis of Tetanus Toxoids as a Quality Control Assay. Vaccines.

[6] Minh A.D., Kamen A.A. (2021). Critical Assessment of Purification and Analytical Technologies for Enveloped Viral Vector and Vaccine Processing and Their Current Limitations in Resolving Co-Expressed Extracellular Vesicles. Vaccines.

[7] Chen K., Li C., Wang Y., Shen Z., Guo Y., Li X., Zhang Y. (2021). Optimization of Vero Cells Grown on a Polymer Fiber Carrier in a Disposable Bioreactor for Inactivated Coxsackievirus A16 Vaccine Development. Vaccines.

[8] Imai T. (2021). Single Amino Acid Deletion at N-Terminus of the Target Antigen in DNA Vaccine Induces Altered CD8(+) T Cell Responses against Tumor Antigen. Vaccines.

[9] Martinez-Perez A.G., Perez-Trujillo J.J., Garza-Morales R., Ramirez-Avila N.E., Loera-Arias M.J., Gomez-Gutierrez J.G., Saucedo-Cardenas O., Garcia-Garcia A., Rodriguez-Rocha H., Montes-de-Oca-Luna R. (2021). An Oncolytic Adenovirus Encoding SA-4-1BBL Adjuvant Fused to HPV-16 E7 Antigen Produces a Specific Antitumor Effect in a Cancer Mouse Model. Vaccines.

